# Bacterial communities associated with an island radiation of lichen-forming fungi

**DOI:** 10.1371/journal.pone.0298599

**Published:** 2024-03-18

**Authors:** Miguel Blázquez, Rüdiger Ortiz-Álvarez, Francisco Gasulla, Israel Pérez-Vargas, Sergio Pérez-Ortega

**Affiliations:** 1 Real Jardín Botánico, CSIC, Madrid, Spain; 2 Fundación Española para la Ciencia y la Tecnología (FECYT), Alcobendas, Spain; 3 Department of Life Sciences, Universidad de Alcalá, Alcalá de Henares, Spain; 4 Department of Botany, Ecology and Plant Physiology, Universidad de La Laguna, San Cristóbal de La Laguna, Spain; Friedrich Schiller University, GERMANY

## Abstract

Evolutionary radiations are one of the most striking processes biologists have studied in islands. A radiation is often sparked by the appearance of ecological opportunity, which can originate in processes like trophic niche segregation or the evolution of key innovations. Another recently proposed mechanism is facilitation mediated by the bacterial communities associated with the radiating species. Here we explore the role of the bacterial communities in a radiation of lichen-forming fungi endemic to Macaronesia. Bacterial diversity was quantified by high throughput sequencing of the V1–V2 hyper-variable region of 172 specimens. We characterized the taxonomic and phylogenetic diversity of the bacterial communities associated with the different species, tested for compositional differences between these communities, carried out a functional prediction, explored the relative importance of different factors in bacterial community structure, searched for phylosymbiosis and tried to identify the origin of this pattern. The species of the radiation differed in the composition of their bacterial communities, which were mostly comprised of Alphaproteobacteria and Acidobacteriia, but not in the functionality of those communities. A phylosimbiotic pattern was detected, but it was probably caused by environmental filtering. These findings are congruent with the combined effect of secondary chemistry and mycobiont identity being the main driver of bacterial community structure. Altogether, our results suggest that the associated bacterial communities are not the radiation’s main driver. There is one possible exception, however, a species that has an abnormally diverse core microbiome and whose bacterial communities could be subject to a specific environmental filter at the functional level.

## Introduction

Oceanic islands, those which have never been connected to continental landmasses, have attracted the interest of naturalists and evolutionary biologists since the 19th century. Their isolation and the smaller size of their biotas compared to continental landmasses make them ideal natural laboratories for studying the origin and evolution of biodiversity [1,2]. One of the most studied evolutionary phenomena on oceanic islands are evolutionary radiations, that is, how a single colonization event diversifies by cladogenesis into a collection of species [3]. Radiations can be divided in adaptive and non-adaptive [4–6]. In non-adaptive radiations, diversification is not accompanied by niche differentiation and gives rise to ecologically similar allopatric species [4,6]. In contrast, adaptive radiations are those in which ecological speciation drives diversification, producing ecologically different species, either allopatric or sympatric [5]. In adaptive radiations species differ in traits which show clear correlations with the niche they occupy [5,7]. Hence, when studying the adaptive nature of a radiation, it is essential to explore the variability of functional traits in the species and their correlation with abiotic factors. Textbook examples of adaptive radiation have focused on flagship organisms such as the silversword alliance of Hawaii, that have diversified colonizing a vast array of habitats including rain forests, bogs and alpine regions [8]. Silversword species are varied in their physiology and morphology (e.g. growth form, rates of transpiration, leaf shape, etc.), and these variations are correlated with the habitats in which the species are found [9–11]. Putative radiations have also been reported in less studied organisms [12–14], but their adaptive nature has rarely been studied [15,16].

Although the study of adaptive radiations has usually focused on the morphological variability of traits presumably related to the performance of the species in a certain environment [17,18], or on physiological traits that denote differences in the use of resources associated with certain niches [19], Gillespie *et al*. [20] proposed that symbiotic interactions with microbes could facilitate adaptive radiation. The rationale behind this hypothesis is that specific microbiomes may mobilize previously unobtainable nutrients and harness new forms of energy (e. g. [21]), helping their hosts colonize an ecological space that may otherwise be unsuitable for them. The role of these interactions on adaptive radiations could be particularly relevant for lichens, a symbiosis between a fungus and different types of microorganisms (i.e., algae, cyanobacteria, other fungi, and various microbes). The bacterial communities of the lichen thallus are implicated in numerous functions [22,23]: nutrient supply, including nitrogen fixation and production of aminoacids and vitamins [24–27], growth hormone production [28] and resistance against abiotic factors [22], pathogens [29] and toxins [30].

Bacterial communities of closely related species are generally more similar than those of more distantly related species [31], and can even recapitulate the phylogeny of their hosts. Such pattern has been termed phylosymbiosis [32] and has been found in plants [33], spiders [34] and primates [35] but, to our knowledge, it has never been studied in lichen-forming fungi. The mechanisms by which phylosymbiosis emerge are not clear [36,37], although two agreed hypothesis are the vertical transmission of microbial communities [38,39] and environmental filtering [36,40]. The role of associated microbiomes in adaptive radiations has been at least partially explored in some of the best studied radiations, such as in the Darwin finches [41–43], the *Anolis* lizards [44] or the African cichlid fishes [45], but so far its role in radiations of lichen-forming fungi remains unexplored.

One of the most striking examples of radiation in lichen-forming fungi is the genus *Ramalina* in the islands of Macaronesia [46]. *Ramalina* has a subcosmopolitan distribution, but most of its diversity occurs in five regions: East Africa [47], Baja California [48,49], The Andes [50], Australasia [51,52] and Macaronesia [53–55]. Macaronesia is a biogeographical region formed by a group of volcanic archipelagos in the North Atlantic; Azores, Madeira, Selvagens, Canary Islands and Cape Verde, as well as by a swath of land in continental Africa known as the Macaronesian Enclave [56]. Among the Macaronesian *Ramalina*, two clades of saxicolous species seem to have diversified in parallel, the *R*. *bourgaeana* and the *R*. *decipiens* groups [46,53]. The taxonomy of the *Ramalina decipiens* group has been recently thoroughly studied following an integrative approach [57]. Interestingly, most species are single-island endemics, but two (*R*. *decipiens* and *R*. *maderensis*) are widespread. All are endemic to Macaronesia, with the exception of *R*. *maderensis*, which has been reported from St Helena [58]. This solid taxonomic background and the endemic character of the group makes it and excellent model system to study evolutionary radiations in lichen-forming fungi on oceanic islands (e.g. [16]).

On the basis of the many functions that the associated bacterial communities fulfil in the lichen symbiosis and the fact that these communities can vary between species [23,59–61], we hypothesize that microbiomes may have played an important role in lichen-forming fungi radiations in oceanic islands. To test this hypothesis in the *Ramalina decipiens* group, we specifically address the following questions: (i) does the microbiome composition vary among the species of the group? (ii) Are there differences in the functional capacities of the bacterial communities associated with each species? (iii) Which factors drive microbiome diversity and composition? (iv) Is there a phylosymbiotic pattern? And, if so, (v) is the phylosymbiosis the result of vertical transmission or of environmental filtering?

## Materials and methods

### Sampling

We studied 172 specimens of the *R*. *decipiens* group representing the 15 species-level lineages found by Blázquez *et al*. [57]. Seven of them correspond to previously known species (Krog and Østhagen 1980a; Krog 1990), six to the species that will be described in Blázquez *et al*. [57], here referred to as *R*. *delicata* nom. Prov., *R*. *fortunatarum* nom. Prov., *R*. *gomerana* nom. Prov., *R*. *papyracea* nom. Prov., *R*. *sabinosana* nom. Prov., and *R*. *sampaioana* nom. Prov., and two to undescribed lineages, which are here referred to as *Ramalina* sp. 1 and *Ramalina* sp. 2. The specimens were collected in 48 localities of the seven main Canary Islands, the islands of Sal and São Vicente of the Cape Verde archipelago and the islands of Madeira and Porto Santo of the Madeira archipelago (S1 Table). The number of samples belonging to the different species was proportional to their distribution ranges, with the widely distributed species (*Ramalina maderensis* and *R*. *decipiens*) having more samples than the rest. Also, due to conservation concerns, we collected fewer thalli belonging to the micro-endemic species, as these only occur in one or a few very close localities and their local abundance is not always high. Thalli were collected using forceps that had been sterilized with ethanol. Samples were immediately placed on sterile bags, dried and frozen at -20°C until DNA extraction. Specific permissions were not required, since none of the species in the *R*. *decipiens* group are listed as protected species in any official document. Specimen collection in natural protected areas was authorized by Secretaria Regional do Ambiente e Recursos Naturais of the Governo Regional da Madeira, Parque Nacional de Garajonay, Cabildo Insular de El Hierro, Cabildo Insular de Tenerife, Cabildo Insular de Gran Canaria and Cabildo Insular de Lanzarote.

### DNA extraction, amplification and sequencing

Approximately 20 mg of lichen material were selected for DNA extraction under a Nikon SMZ800 stereomicroscope with the help of razor blades and forceps. Tools were sterilized between samples using ethanol. Entire laciniae were included in order to capture bacterial diversity associated with different zones of the thalli [23]. Laciniae showing conspicuous biofilms or those colonized by lichenicolous fungi were excluded. Samples were then placed into Eppendorf tubes and stored at −80ºC. Frozen samples were pulverized using TissueLyser II (Qiagen) with two crystal beads prior to DNA isolation. Genomic DNA was extracted using the PowerSoil DNA Isolation Kit (MO BIO Laboratories, Carlsbad, CA, United States), following the instructions of the manufacturer. The V1–V2 hyper-variable region of the bacterial 16S rRNA was used as barcode to prospect bacterial diversity. We used universal primers 27F and 338R [62,63] for amplification. PCR reactions were carried out in a total volume of 15 μl, containing 3 μl of template DNA, 1 μl of each primer (10 μM), 7 μl of ACCUZYME^™^ DNA Polymerase Mix, 2x (Bioline, Sydney, Australia) and 3 μl of distilled water. PCR settings consisted in an initial denaturation at 95ºC for 5 min; 30 cycles of 94ºC for 1 min, 54ºC for 1 min and 72ºC for 1 min; with a final extension at 72ºC for 7 min. PCR products were checked in 1% agarose gels stained with SYBR^™^ Safe DNA Gel Stain (Thermo Fisher Scientific) and quantified using the Qubit dsDNA HS (High Sensitivity) Assay Kit (Thermo Fisher Scientific). Then they were pooled in equimolar concentrations and sequenced on a single MiSeq run (Illumina, USA) using v2 chemistry and 2 × 250-bp paired-end reads at the RTSF Genomics Core at Michigan State University (East Lansing, Michigan).

### Sequence processing

Raw sequences were processed using DADA2 [64] in R 4.1.1 [65] using the parameters described in Callahan *et al*. [66]. In short, DADA2 takes a set of demultiplexed paired-end fastq files, filters the sequences based on their quality and length and assembles them into amplicon sequence variants (ASVs) taking sequencing errors into account. We removed chimeric ASVs and assigned taxonomy to the remaining ones with the functions *assignTaxonomy* and *addSpecies* of DADA2 using the SILVA 132 database [67] as reference. We generated rarefaction curves using the *rarecurve* function of the *vegan* R package [68]. All sequences obtained in this study are available in the SRA (NCBI) under BioProject PRJNA973550. We obtained 8,442,731 raw reads (min = 953, mean = 46,937 and max = 70,066), of which 1,976,254 passed DADA2 quality filter (min = 101, mean = 11,228 and max = 39,279) and clustered into 3,920 ASVs. After removing ASVs corresponding to chloroplasts and mitochondria, those that were not identified as Bacteria in the SILVA database, and those present in less than 1% of the samples 1,798,461 reads belonging to 586 ASVs remained. Rarefaction curves indicated that a sequencing depth of 2,000 reads was enough to capture the bacterial communities of the thalli (S1 Fig). 33 samples belonging to seven species (*R*. *decipiens*, *R*. *erosa*, *R*. *fortunatarum*, *R*. *hamulosa*, *R*. *maderensis*, *R*. *nematodes* and *R*. *sabinosana*) were removed because their sequencing depth was below this threshold. An additional sample (M134) was also dropped because it harboured an abnormally large number of ASVs. Thus, the filtered dataset included 138 samples belonging to the 15 species of the group.

### Alignment and phylogenetic relationships

We aligned the remaining ASVs using SINA [69] and removed the hypervariable regions in Gblocks [70,71] selecting settings for the least stringent selection available (ran on a webserver: http://molevol.cmima.csic.es/castresana/Gblocks_server.html). In order to mitigate the uncertainty derived of using short sequences for phylogenetic inference we constrained the tree topology at the phylum and class levels to reflect ASV taxonomy, based on the SILVA database. The phylogenetic analysis was carried out in BEAST 1.8.1 [72,73]. We used a lognormal uncorrelated relaxed clock and the substitution model GTR+I+G. The Tree Prior “Speciation: Birth-Death” was selected. A randomly generated tree was used as the starting point. The analysis was set to run for 2 x 10^8^ generations sampling every 20,000 steps. The phylogenetic tree was time calibrated using secondary calibration, implemented with normal priors to calibrate the nodes. We established seven calibration points at the phylum level and two at the class level based on the crown ages reported by Marin *et al*. [74] for the origin of Acidobacteria (2,750 Mya), Actinobacteria (1,400 Mya), Alphaproteobacteria (1,850 Mya), Bacteroidetes (1,500 Mya), Cyanobacteria (2,100 Mya), Firmicutes (2,400 Mya), Fusobacteria (2,500 Mya), Gammaproteobacteria (1,700 Mya) and Planctomycetes (1,650 Mya). The sampled population of trees was processed with TreeAnnotator v.1.8.1 (http://beast.bio.ed.ac.uk/treeannotator) with a 50% burnin to generate an annotated maximum clade credibility tree. The post-burnin population of 5,000 trees was kept to take phylogenetic uncertainty into account in downstream analyses. The BEAST analysis was performed in the Trueno cluster facility of the SGAI-CSIC.

### Microbiome characterization

We combined the ASV table, the taxonomy and the phylogenetic tree of the ASVs in a single phyloseq [75] object. Taxonomic profiles of the bacterial community of each sample were produced at the class level using the functions *transform*, *aggregate_rare* and *plot_composition* on the raw phyloseq object in the *microbiome* R package (http://microbiome.github.io). ASVs forming the strict (i. e. ASVs appearing in all the samples of a given species, [76]) and relaxed (i. e. ASVs appearing in at least half the samples of a given species) core microbiomes of the species were assessed using the *core* function of the *microbiome* R package for those species represented by at least four samples. For visualization we produced a heatmap with the function *plot_heatmap* of the *phyloseq* R package. Alpha-diversity metrics, more specifically ASV richness, inverse Simpson diversity, Shannon diversity, Pielou’s Evenness, Faith’s phylogenetic diversity (PD, [77]) and the standardized effect size of PD (sesPD) were calculated using the function *alpha* of the *microbiome* R package and *pd* and *ses*.*pd* of the *picante* R package [78]. sesPD was calculated with the null model "taxa.labels" and 9,999 permutations. Richness, PD and sesPD were calculated from a rarefied phyloseq object that was generated using the function *rarefy_even_depth* of the *phyloseq* R package. Inverse Simpson diversity, Shannon diversity and Pielou’s Evenness were calculated from a phyloseq object in which we transformed the raw ASV abundance into relative abundance using the function *phyloseq_standardize_otu_abundance* of the *metagMisc* R package [79] with the method "total". To take phylogenetic uncertainty into account, PD and sesPD were calculated for 100 randomly selected trees of the post-burnin population. To test the statistical significance of the observed differences we computed an ANOVA followed by a post-hoc Nemenyi’s non-parametric all-pairs comparison test for each of the indices using the functions *aov* of the *stats* R package [65] and *kwAllPairsNemenyiTest* of the *PMCMRplus* R package [80], respectively. In order to explore if the composition of the bacterial communities was different across the *Ramalina* species a non-metric multidimensional scaling (NMDS) ordination based on the Bray-Curtis distance was computed using the functions *transform_sample_counts* and *ordinate* of the *phyloseq* R package. The same approach was used to explore compositional differences in the ASVs forming the relaxed core microbiomes of the *Ramalina* species represented by at least four samples. The statistical significance of the observed differences was tested by analyses of similarities (ANOSIM, [81]) using the *anosim* function of the *vegan* R package with 9,999 permutations. Additionally, we used PERMDISP [82], implemented in the function *betadisper* of *vegan*, to determine if the dispersion of the whole bacterial communities and the ASVs forming the relaxed core microbiomes differed across species. The phyloseq object with ASV relative abundance was used to compute the NMDS, ANOSIM and PERMDISP.

### Functional prediction

To explore the functionality of the bacterial communities associated with the different species of the group, we used a functional prediction implemented in PICRUST2 [83]. Briefly, PICRUST2 places ASVs into a reference genomic tree of 20,000 bacterial and archaeal species [84] and uses this information to predict the ASVs genomes. The functional prediction is derived from the gene-family copy numbers assigned to each ASV. To summarize the PICRUST2 output, first, we identified the metabolic pathways in which the species differed significantly. This was done by ANOVA in Statistical Analysis of Metagenomic Profiles (STAMP, [85]). Only the species that were represented by more than two specimens were used in this step. The remaining species were dropped from the following analysis. The values of these pathways for every specimen were then standardized and included in a principal component analysis (PCA), which was computed using the *prcomp* function in the *stats* R package [65]. The coordinates of the specimens in the first two principal components were extracted and used as synthetic trait values. Finally, these two synthetic values were used to explore interspecific differences in bacterial functionality. This was done by ANOVA, which was computed using the *aov* function of the *stats* R package.

### Factors driving microbiome community structure

The relative importance of different factors over the structure of the bacterial communities was explored by distance-based redundancy analysis (dbRDA, [86]). We calculated an abundance-weighed phylogenetic UniFrac distance-based dissimilarity matrix [87] in the *phyloseq* R package. We used a phyloseq object with ASV relative abundances, calculated using the function *phyloseq_standardize_otu_abundance* of the *metagMisc* R package, to compute the dissimilarity matrix using the function *UniFrac* of the *phyloseq* R package. The dissimilarity matrix was used as the response matrix for the dbRDA. We used four explanatory matrices: 1) the first matrix corresponded to the islands where the samples were collected, 2) the second consisted in the *Ramalina* species to which they belonged, 3) the third corresponded to the main lichen substance present in the thalli (salazinic acid, lecanoric acid, protocetraric acid, divaricatic acid or 4-O-demetilbarbatic acid) and 4) the fourth included macroclimate information based on five variables downloaded from WorldClim [88]: precipitation (mm), average temperature (°C), wind speed (m s^-1^), solar radiation (kJ m^-2^ day^-1^) and water vapour pressure (kPa). As in previous studies [16], we used these variables instead of the commonly used bioclim variables because those are solely based on temperature and rainfall and do not include information on other factors that are important to lichens, such as solar radiation or water vapour pressure. We downloaded all layers of each variable (one for every month of the year) at a resolution of 30 seconds (~1 km^2^). Then, they were merged into year averages using the *calc* function of the *raster* R package [89]. Variable values for each sample were calculated based on their geographic coordinates using the function *extract* from the *raster* R package. Prior to the calculation of the dbRDA we checked for correlation between the macroclimatic variables. For this, we calculated a Spearman correlation matrix that was used to produce a correlation hierarchical cluster plot with the absolute correlation values. Precipitation and water vapour pressure were dropped as they were correlated with solar radiation and average temperature, respectively. Lastly, we checked the normality of the remaining variables by visual inspection of their histograms. Histograms were generated with the *hist* function of the *stats* R package [65]. Solar radiation was not normal and was log-transformed. dbRDA was calculated with the *dbrda* function of the *vegan* R package. Variance partitioning between the four explanatory matrices was calculated using adjusted R^2^. Statistical significance of the adjusted R^2^ was assessed for each fraction with a permutation based anova test with 2000 permutations. This was done using the functions *varpart* and *anova*.*cca* of the *vegan* R package. Significance of the dbRDA as a whole was also assessed with a permutation based anova test with 2000 permutations, this time with the function *anova*. The whole analysis was repeated using the phyloseq object containing the relative abundances of the ASVs belonging to the relaxed core microbiomes of the nine *Ramalina* species represented by at least four samples. Venn diagrams were plotted to visualize variance partitioning.

### Assessing phylosymbiosis

We used the Procrustean approach to co-phylogeny (PACo, [90]) to explore if there was a phylosymbiotic pattern between the *Ramalina* species and their microbiomes. PACo was originally conceived to detect co-evolution patterns in host-parasite studies. Here, the bacterial communities are treated as the parasites and are compared with the phylogeny of the *R*. *decipiens* group produced by Blázquez *et al*. [57]. The *Ramalina* phylogeny was transformed into a distance matrix using the *cophenetic* function of the *stats* R package [65]. We used an abundance-weighed phylogenetic UniFrac distance-based dissimilarity matrix, calculated as in previous sections from a phyloseq object with the relative abundance of the ASVs, as the microbiome distance matrix. PACo was ran on these matrices using the functions *prepare_paco_data*, *PACo* and *paco_links* of the *paco* R package [91]. Statistical significance of the analysis was calculated with 10,000 permutations. To take phylogenetic uncertainty into account, both for the *Ramalina* species and their microbiomes, PACo analysis was replicated 100 times with randomly selected trees from the post-burning tree populations of the *Ramalina* species and the bacterial ASVs. The correlation coefficient *r* was calculated as *r* = (1-*ss*).

To further explore the cause of the phylosymbiosis between the *Ramalina* phylogeny and their bacterial communities we carried out beta diversity through time analysis (BDTT, [92]). BDTT samples the bacterial phylogeny at user defined time intervals and provides a correlation profile between the diversification of the bacterial taxa and a user defined variable for each time interval. This methodology was developed to try to differentiate between several processes that could generate congruence between the phylogeny of a host and its associated bacterial communities—co-evolution of both partners or phylogenetically correlated environmental filters. A BDTT profile showing high correlation coefficients at recent times and low correlation further back in evolutionary time is indicative of recent bacterial diversification being responsible for the congruence between the variable and the bacterial communities. Alternatively, BDTT profiles showing high correlation back in time indicate that ancient bacterial lineages are driving the observed correlation. It is important to note that the BDTT profiles expand far beyond the diversification of the *Ramalina decipiens* group itself (around 3 Mya, [46]). Hence, BDTT can be used to discern if the putative correlation between the bacterial phylogeny and the *Ramalina* species is explained by more ancient or more recent bacterial evolution. The former would point to environmental filtering, while the later to co-evolution. We calculated BDTT as described in Groussin *et al*. [92]. Briefly, we sliced the bacterial phylogeny in units of 10 Mya and computed BDTT using a phyloseq object with the relative abundance of the ASVs, which we obtained with the function *phyloseq_standardize_otu_abundance* of the *metagMisc* R package. This was done using the R function *BDTT* provided in the BDTT original paper [92] using the Bray-Curtis distance. Then, we calculated the correlation between BDTT and three explanatory variables (mycobiont identity, island and chemistry) through PERMANOVA. This was done using the *adonis* function of the *vegan* R package.

## Results

### Microbiome characterization

**Fig 1:**
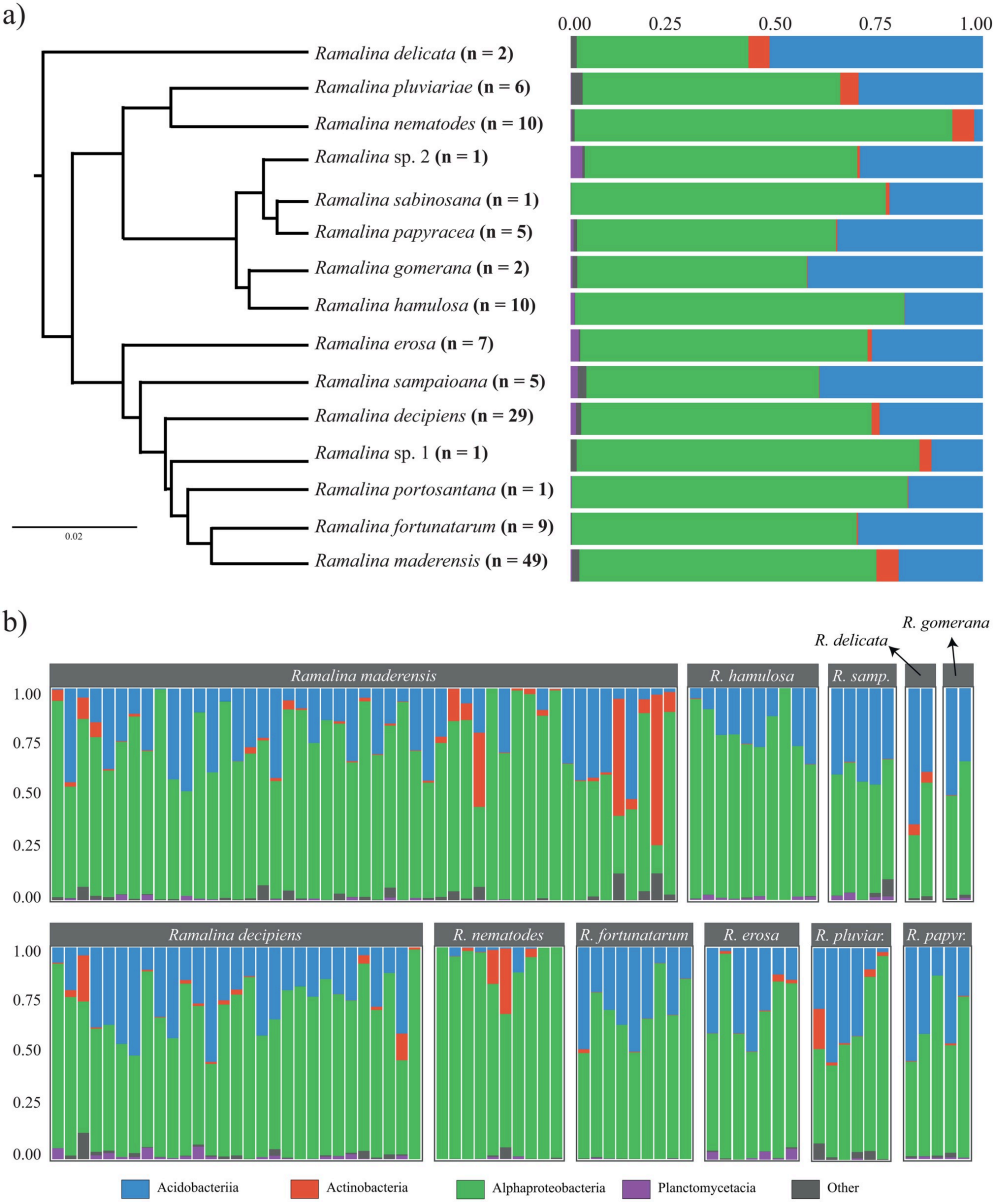
Taxonomic profiles at class level of the bacterial communities found in the *Ramalina decipiens* group species. A) Mean relative abundance of the bacterial classes across all samples of each species adjoining an ultrametric phylogenetic tree depicting the relationships between them (modified from Blázquez *et al*. [57]). The number of samples of each species is depicted alongside its name. B) Relative abundance of the bacterial classes in individual thalli, grouped by species.

**Fig 2:**
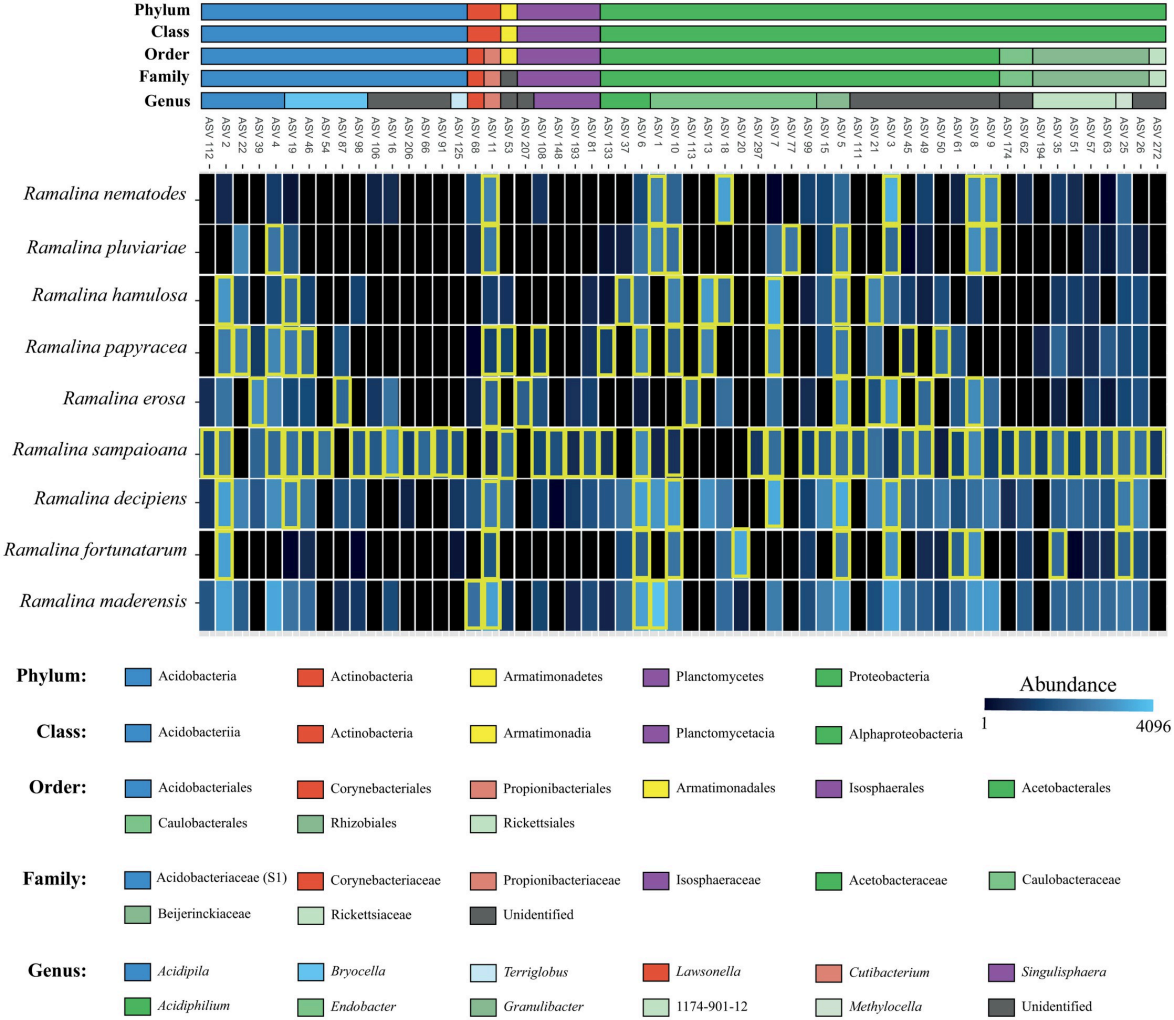
Heatmap depicting the taxonomy and abundance of the bacterial ASVs that comprised the relaxed core microbiome (i. e. ASVs appearing in at least half the samples) of the nine *Ramalina* species represented by more than four samples. ASVs belonging to the core microbiome of each species are highlighted in yellow.

The 586 ASVs belonged to 20 classes (Fig 1), the most common being Alphaproteobacteria (71.57% of the reads) and Acidobacteriia (23.35%). 20 ASVs belonging to four classes were found to be part of the strict core microbiome of the nine species represented by at least four samples. Only 4 of these (ASV3, ASV7, ASV8 and ASV11) were part of the core microbiome of more than one species. The median number of ASVs in the strict core microbiomes was 1. The two widespread species, *Ramalina decipiens* and *R*. *maderensis*, did not have strict core microbiomes. On the other hand, *Ramalina sampaioana* nom. Prov. had a strict core microbiome comprised by 13 ASVs, some of them belonging to families not present in the strict core microbiomes of the other species, specifically *Beijerinckiaceae*, *Caulobacteraceae* and *Isosphaeraceae*. 58 ASVs belonging to five classes were found to be part of the relaxed core microbiome of the nine species (Fig 2). No ASV belonged to the core microbiome of all species. All core microbiomes were mostly comprised by ASVs belonging to the families *Acetobacteraceae* (Proteobacteria), *Acidobacteriaceae* (subgroup 1, Acidobacteria) and *Propionibacteriaceae* (Actinobacteria). The median number of ASVs in the relaxed core microbiome of the species was 9. The least diverse was that of *Ramalina maderensis*, with just four ASVs. On the other hand, *Ramalina sampaioana* nom. Prov. harboured a relaxed core microbiome comprised by 43 ASVs, many of them belonging to genera not present in the core microbiomes of the other species. These were *Terriglobus* (*Acidobacteriaceae* subgroup 1) and unidentified genera belonging to the families *Acidobacteriaceae* subgroup 1 (Acidobacteria), *Caulobacteraceae* (Proteobacteria), *Beijerinckiaceae* (Proteobacteria) and *Rickettsiaceae* (Proteobacteria).

**Fig 3:**
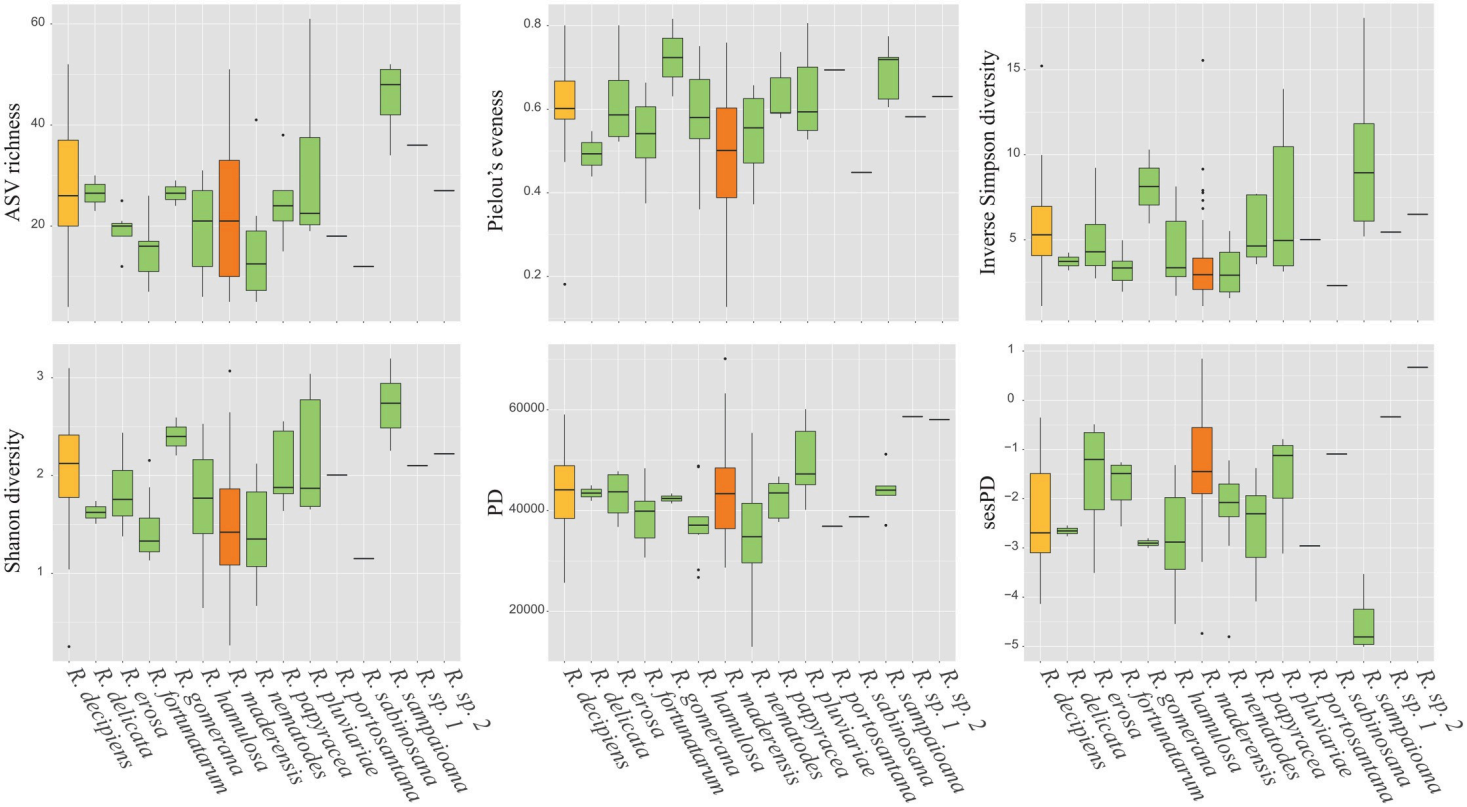
Boxplots representing the six alpha-diversity metrics (ASV richness, inverse Simpson diversity, Shannon diversity, Pielou’s Evenness, PD and sesPD) for each *Ramalina* species. Species occurring in one, two or three archipelagos are shown in green, yellow and orange, respectively.

**Fig 4:**
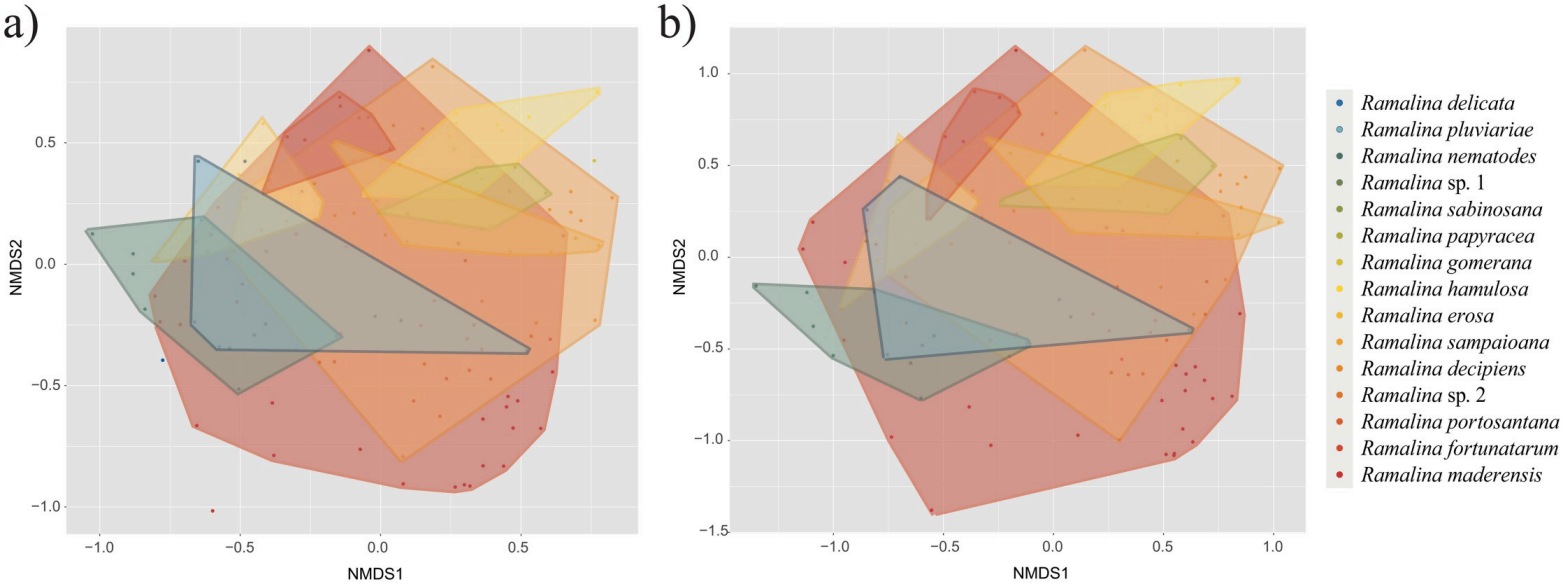
NMDS ordination plots based on Bray-Curtis dissimilarity matrices for (a) the whole bacterial communities and (b) the ASVs belonging to the relaxed core microbiome of the nine *Ramalina* species represented by at least four samples.

We found differences in alpha diversity between the bacterial communities of the *Ramalina* species (Fig 3, Table 1). The post-hoc tests (S2 Table) revealed that this had its origin in differences between *Ramalina sampaioana* nom. Prov. and/or *R*. *maderensis* and other species. Despite ANOVA showing statistically significant differences between species phylogenetic diversity, the post-hoc tests did not find supported differences between them. The NMDS ordination plots (Fig 4) showed differences in the structure of bacterial communities associated to each *Ramalina* species. This pattern is somewhat obscured by the high intraspecific variability of *Ramalina maderensis* and *R*. *decipiens*, the two species of the group with the largest distribution ranges. The pattern was observed analysing the whole bacterial communities (Fig 4a) as well as the relaxed core microbiomes (Fig 4b). The existence of differences in community composition between species was statistically confirmed by ANOSIM results, both for the whole bacterial community (*R* = 0.245, *p* < 0.001) and for the ASVs forming the relaxed core microbiomes (*R* = 0.262, *p* < 0.001). Differences in bacterial community dispersion between *Ramalina* species were statistically confirmed by PERMDISP in the whole communities (*F* = 7.652, *p* < 0.001), and in the relaxed core microbiomes (*F* = 4.633, *p* < 0.001).

**Table 1:** ANOVA results for the alpha diversity indices.

	*F*	*p*
ASV richness	2.968	<0.001***
Pielou’s evenness	3.349	<0.001***
Inverse Simpson diversity	3.539	<0.001***
Shannon diversity	2.787	0.0012**
PD	2.021	0.0212*
sesPD	6.101	<0.001***

*P < 0.05,

**P < 0.01,

***P < 0.001.

### Functional prediction

PICRUST2 identified 342 metabolic pathways in the bacterial communities associated with the *R*. *decipiens* group species (S3 Table). Of these, 185 differed significantly between *Ramalina* species. The first two principal components of the PCA computed with these pathways explained 51.55% and 11.77% of the total variance, respectively. All species overlapped in the two principal components (S2 Fig) although, interestingly, *R*. *sampaioana* nom. Prov. showed less dispersion than the other species. However, ANOVA did not find statistically significant differences in the overall functionality of the bacterial communities, neither in the first (*F* = 1.385, *p* = 0.109) nor in the second (*F* = 1.844, *p* = 0.075) principal components.

### Factors driving microbiome community structure

**Fig 5:**
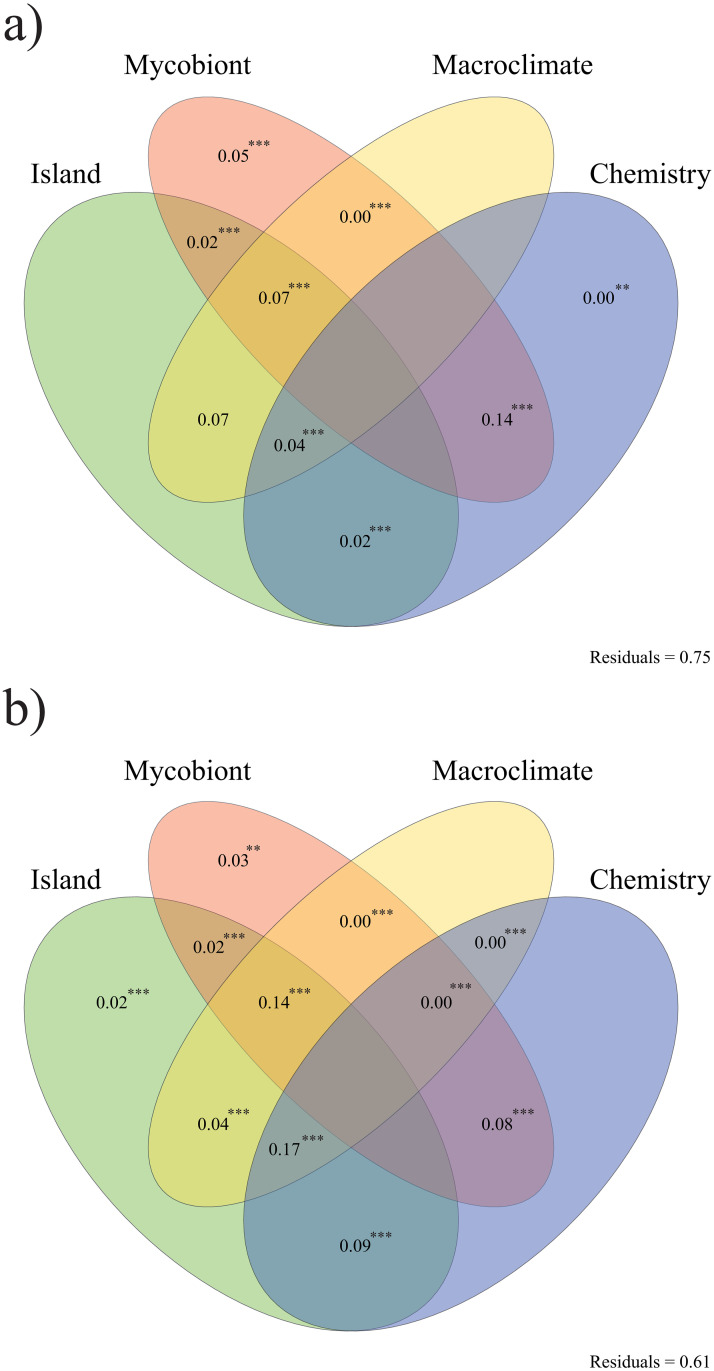
Venn diagrams showing variance partitioning between the combined and simple effects of the variables included in the dbRDAs (chemistry, island, macroclimate and mycobiont identity) over bacterial community structure in (a) the whole communities and (b) the ASVs belonging to the relaxed core microbiome of the nine *Ramalina* species represented by at least four samples. The explained variation indicated are the adjusted R^2^ values. Values < 0 not shown. *P < 0.05, **P < 0.01, ***P < 0.001.

The dbRDA analysis (Fig 5a) explained 33% of variance, showing that the main driver of bacterial community structure was the combined effect of chemistry and mycobiont species (*F* = 1.895 with a *p* = 0.001). Considering the possibility that this pattern was an artefact caused by the chemical variability of *R*. *maderensis*, which shows four different chemotypes and is the most represented species in the study, we repeated the analysis excluding *Ramalina maderensis* and the pattern remained (data not shown). The dbRDA conducted over the standardized phyloseq object containing the ASVs forming the relaxed core microbiome of the species represented by at least four samples returned a *F* = 2.920 with a *p* = 0.001. Its constrained axes explained 38% of the variance in ASV communities. The variance partitioning pattern was different to the one in the former case (Fig 5b). There was not a single driver of bacterial community composition, as the most important effects where those of the combination of two or even three variables. The combined effect of chemistry and mycobiont identity remains, but its importance is surpassed by that of other variables.

### Assessing phylosymbiosis

**Fig 6:**
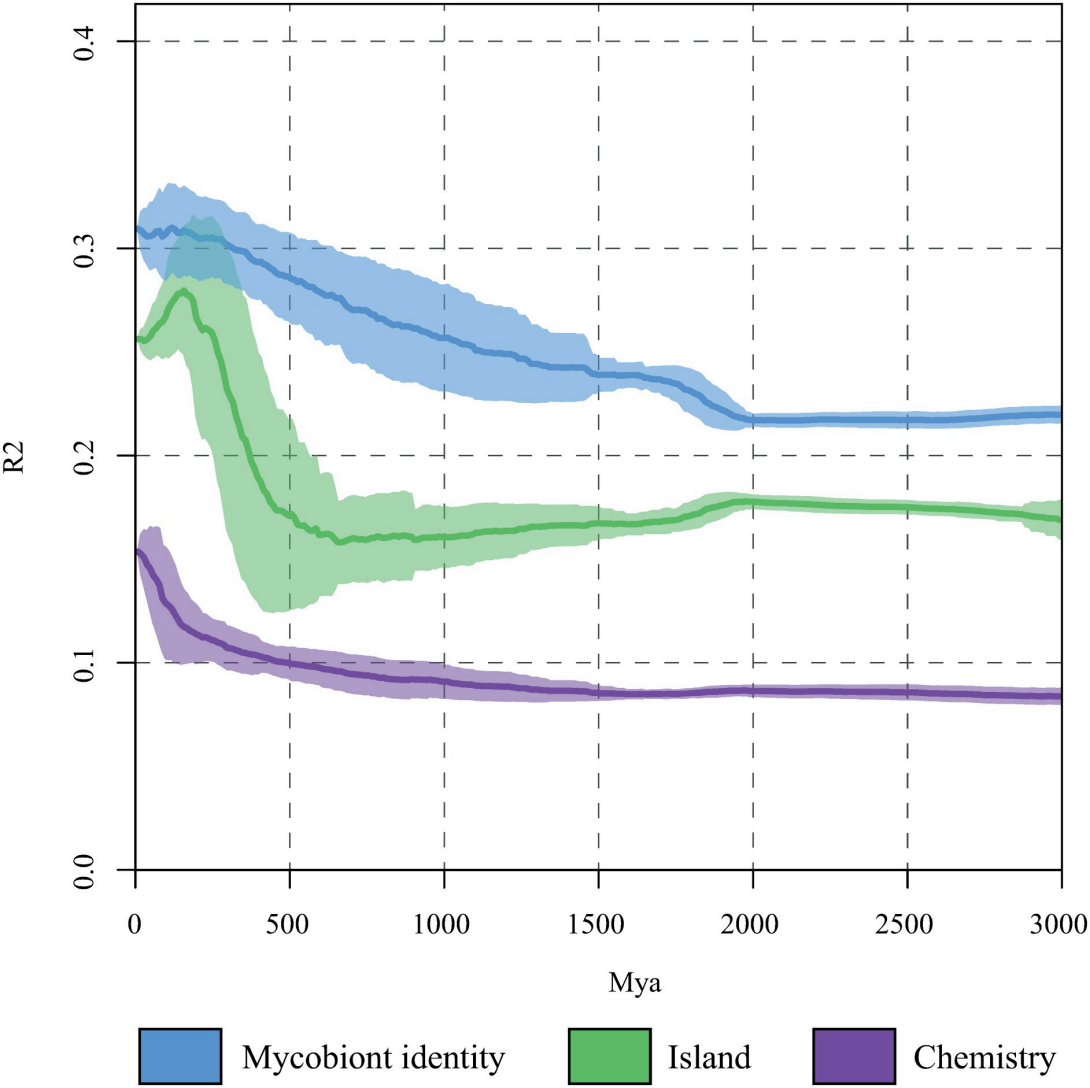
Beta diversity through time analysis. Profiles show the correlation between the pairwise Bray Curtis dissimilarities of the bacterial communities determined by time slices every 10 Mya. Bold lines are the mean values across 100 randomly selected phylogenetic trees and envelopes represent standard deviations.

PACo analysis detected a significant correlation between the phylogenetic distances between *Ramalina* thalli and their microbiome communities (*r* = 0.66 ± 0.10, *p* = 0.020 ± 0.019; values here reported are the means and standard deviations across the 100 replicates). Thus, a phylosymbiotic pattern does exist. BDTT profiles showed that ‘Mycobiont identity’ was the variable that most strongly correlated with the beta diversity (Fig 6). Its correlation coefficient was always above R^2^ = 0.20, and increased steadily to the present, raising above R^2^ = 0.30 around 300 Mya. ‘Island’ was the second most correlated variable, but its profile was different to that of ‘Mycobiont identity’. It kept almost constant R^2^ values c. 0.18 until around 600 Mya, when it spiked and almost reached R^2^ = 0.30 at around 100 Mya. Afterwards it decreased until the present. The BDTT profile for ‘Chemistry’ shows the overall lowest values of R^2^. It kept R^2^ values c. 0.10 until around 100 Mya, when it spiked and reached R^2^ = 0.15 at present dates.

## Discussion

We explored the role of the associated bacterial communities in the radiation of the *Ramalina decipiens* group. According to our knowledge, this is the first time that the associated bacterial communities have been analysed within the evolutionary framework of an island radiation in lichen-forming fungi. The microbiomes of the 15 species of the group were dominated by Alphaproteobacteria and, to a lesser extent, by Acidobacteriia. Alphaproteobacteria is the dominant bacterial class in many genera of lichen-forming fungi, such as *Cladonia* [93], *Lobaria* [22], *Parmelia* [94], *Peltigera* [95], *Rhizoplaca* [94] or other *Ramalina* species [96]. The fact that Acidobacteriia is the second most abundant bacterial class in the *R*. *decipiens* group is in agreement with previous studies that found that the microbiomes of some saxicolous lichens, such as *Ophioparma* [60] and *Umbilicaria* [97], were dominated by this class. Also, some specimens of *R*. *decipiens*, *R*. *maderensis*, *R*. *nematodes* and *R*. *pluviariae* harboured a significant proportion of Actinobacteria, that even became the dominant class in some of them. A relatively high presence of Actinobacteria has also been reported in *Peltigera frigida* [95] and the bacterial communities of the intertidal *Hydropunctaria maura* are dominated by this class [98]. Actinobacteria are known for their antimicrobial activity [99,100], and some species isolated from lichens are able to produce bioactive compounds (e.g. [29,101,102]), which suggest that Actinobacteria may have a role in defence against pathogens or in the balance of bacterial communities within the thallus [61].

We found highly reduced or even non-existent strict core microbiomes and reduced relaxed core microbiomes associated with the species of the *R*. *decipiens* group. While some lichens harbour a relatively stable core microbiome (e. g. *Lobaria pulmonaria*, [103]), others do not (e. g. *Cladonia stellaris*, [104]). These differences could be related with the predominant reproductive strategy. Aschenbrenner *et al*. ([105]) discovered that the asexual propagules of *Lobaria pulmonaria* host bacterial communities largely similar to those of whole thalli. They argued that these bacteria acted as a ‘starter community’ in the development of the thallus microbiome, which could be linked with the fact that distant *Lobaria pulmonaria* populations share a core microbiome. The asexual propagules of *Lobaria pulmonaria* are isidia and/or soredia, which contain both fungal and algal cells, whereas in most of the *Ramalina decipiens* group species asexual reproduction is mediated by conidia, which are asexual spores of the fungus. This difference in the type of asexual propagules, together with the fact that many of the species in the *R*. *decipiens* group reproduce sexually, could explain the fact that their core microbiomes are formed by a rather low number of ASVs. This result would be congruent with the fact that species in which the co-dispersion of both symbionts is predominant tend to show higher levels of specialization towards their photobionts than sexually reproducing species [106], although the environment may modulate the relationships [107]. The strict and relaxed core microbiomes were comprised by ASVs belonging to the families *Acetobacteraceae* (*Endobacter*, *Acidiphilum* and an unidentified genus) and *Acidobacteriaceae* subgroup 1 (*Acidipila* and *Bryocella*). This could be related with the substrate on which the species of the *R*. *decipiens* group occur: acidic volcanic lavas. Bates *et al*. ([94]) found that *Acetobacteraceae* were fairly dominant in species of *Parmelia*, *Rhizoplaca* and *Umbilicaria* collected from acidic rocks. *Acidobacteriaceae* occur in all manner of acidic environments, from *Sphagnum* peat to uranium-contaminated soils [108]. It would be valuable to carry out a further study to assess the influence of substrate composition on the structure of the bacterial communities associated with these species.

Regarding the functional prediction, we did not find significant differences in the variability of the overall bacterial functionality associated with the *R*. *decipiens* group species, despite the existence of differences in individual metabolic pathways. Such result could be due to the phenomenon of functional redundancy, where communities have similar functional spaces [109,110]. However, the case of *R*. *sampaioana* nom. Prov. is intriguing. This species harbours the most diverse core community of the group, containing ASVs belonging to genera that are not present in the core communities of the other species and, despite ANOVA did not find differences between its microbiome functionality and those of the other species, *R*. *sampaioana* shows a more reduced dispersion in the PCA. This would imply that the functional space of the *R*. *sampaioana* microbiome is particularly homogeneous, perhaps being subject to a specific environmental filter at the functional level. However, since we predict overall metabolic potential but not actual activity, we cannot rule out functional diversity that we have not been able to quantify in our study, requiring to obtain metagenomic, proteomic, or metatranscriptomic data (see [22]). It would also be interesting to characterize the functionality of the bacterial communities of different zones of the thalli and to try to differentiate between ectolichenic and endolichenic bacteria, as previous research showed that there were differences for both variables in *Ramalina farinacea* [96].

We found differences in the composition of the bacterial communities associated with each species of the group, as well as in all the alpha diversity indices. ‘Mycobiont species’ was the only variable that had a simple effect on the structure of the bacterial communities and, together with ‘secondary chemistry’, were the main drivers of community structure. Also, ‘island’ and ‘macroclimate’ influenced the structure of the bacterial communities only through their combined effect with chemistry and mycobiont species. Alonso-García and Villarreal Aguilar ([104]) found that geography was the key factor behind the structure of the bacterial communities associated with *Cladonia stellaris*. Likewise, Cardinale *et al*. ([26]) found that the diversity of Alphaproteobacteria associated with *Lobaria pulmonaria* was mainly explained by geography, while the diversity of *Burkholderia* and the nitrogen fixers was explained by the local habitat. The preponderant role of geography has also been reported by other authors [60,111]. However, it is likely that the effect on community composition exerted by intraspecific differences among populations of the same species cannot be compared with the effect produced by different species.

A plethora of studies have shown unequivocally that the secondary metabolites produced by plants affect the composition and function of their associated microbiomes [112–118]. We have found that the combined effect of secondary chemistry and mycobiont species was the most important driver of bacterial community structure in the *R*. *decipiens* group. These findings are in agreement with Grube and Berg [119], who argued that differences in chemistry between lichens, together with thallus structure, could create different ecological niches for the associated microorganisms. To our knowledge, this is the first time that the relative importance of secondary chemistry in shaping the bacterial communities associated with lichen thalli has been explicitly explored, in spite of the well-known role of certain secondary metabolites as potential antibiotics [120,121]. Also, the species of the *Ramalina decipiens* group produce compounds such as depsides and depsidones [53] that are known to have antimicrobial activity, like divaricatic acid [122], protocetraric acid [123] and salazinic acid [124], which further points to secondary metabolites having an important role in shaping the associated microbiomes.

We detected a phylosymbiotic pattern between the *Ramalina* phylogeny and the bacterial communities associated with the thalli. However, the high correlation values in the beta diversity through time profiles (around 100 Mya), well beyond the divergence time of the *Ramalina decipiens* group (around 3 Mya, [46]), suggest that this pattern is due to divergence among ancient lineages of bacteria, which points to phylogenetically structured environmental filtering and does not support the co-evolution hypothesis. These findings are congruent with those reported by Loo *et al*. ([42]) for the Darwin finches, which also showed phylosymbiosis and BDTT profiles indicative of environmental filtering. They are also congruent with those found by Perez-Lamarque *et al*. ([125]) for the Hawaiian *Ariamnes* spiders, who show a significant phylosymbiosis with their microbiota that is most likely not caused by vertical transmission. The relationship between microbiomes and host diversification has been explored in other well-known examples of adaptive radiations, namely the *Anolis* lizards [44] and the African and American cichlid fishes [126], although the existence of phylosymbiosis has not been explicitly tested. In both cases the host phylogeny was related to the associated bacterial communities, but weakly. Ren *et al*. ([44]) found substantial variability in the microbiomes associated with the different *Anolis* species and ecomorphs (i. e. species that occupy the same niche and are similar in morphology and behaviour), but there were no significant differences in alpha diversity among them. They did find subtle differences in beta diversity related to host phylogeny, but not to the ecomorphs. The cichlid microbiomes were firstly influenced by geography (continent and lake) and then by genetic and ecological distances between species [126]. This could be related with the fact that all these are rather recent radiations. Host-microbiome co-diversification has been detected in older groups, like mammals [92] or sea turtles [127].

## Conclusions

We have found that the species of the *Ramalina decipiens* group differ in the composition of their associated bacterial communities, that mycobiont identity is an important driver of the bacterial community structure and that a phylosymbiotic pattern between the *Ramalina* species and their microbiomes does exist. However, the main driver of bacterial community structure is the combined effect of secondary chemistry and mycobiont identity, the phylosymbiotic pattern is most likely caused by phylogenetically structured environmental filtering and the bacterial communities associated with the different species do not show overall functional differences. Based on these findings we discard a prominent role of the bacterial communities associated with the *Ramalina decipiens* group in its diversification within Macaronesia, but they could have had a more relevant role in the origin of one species, *Ramalina sampaioana*. This hypothesis should be properly tested from a multi-omics perspective.

## Supporting information

S1 Fig Rarefaction curves. (PDF)

S2 Fig Overall functional diversity of the bacterial communities associated with the *R*. *decipiens* group species. Boxplots show the coordinates of the studied specimens in the (a) first and (b) second principal components of the PCA computed from the 185 metabolic pathways predicted by PICRUST2 in which there where interspecific differences.
(PDF)

S1 Table Metadata containing DNA isolate codes, species identification, specimen collector numbers, geographic origin, secondary chemistry and coordinates. (XLSX)

S2 Table Post-hoc Nemenyi’s non-parametric all-pairs comparison tests for each of the alpha diversity indices. (XLSX)

S3 Table PICRUST2 metabolic pathways. (XLSX)
