# Supplementary figures and images for: Bacterial communities associated with an island radiation of lichen-forming fungi
Source: PLoS One. 2024 Mar 18;19(3):e0298599. doi: 10.1371/journal.pone.0298599 (PMC10947700; doi:10.1371/journal.pone.0298599)

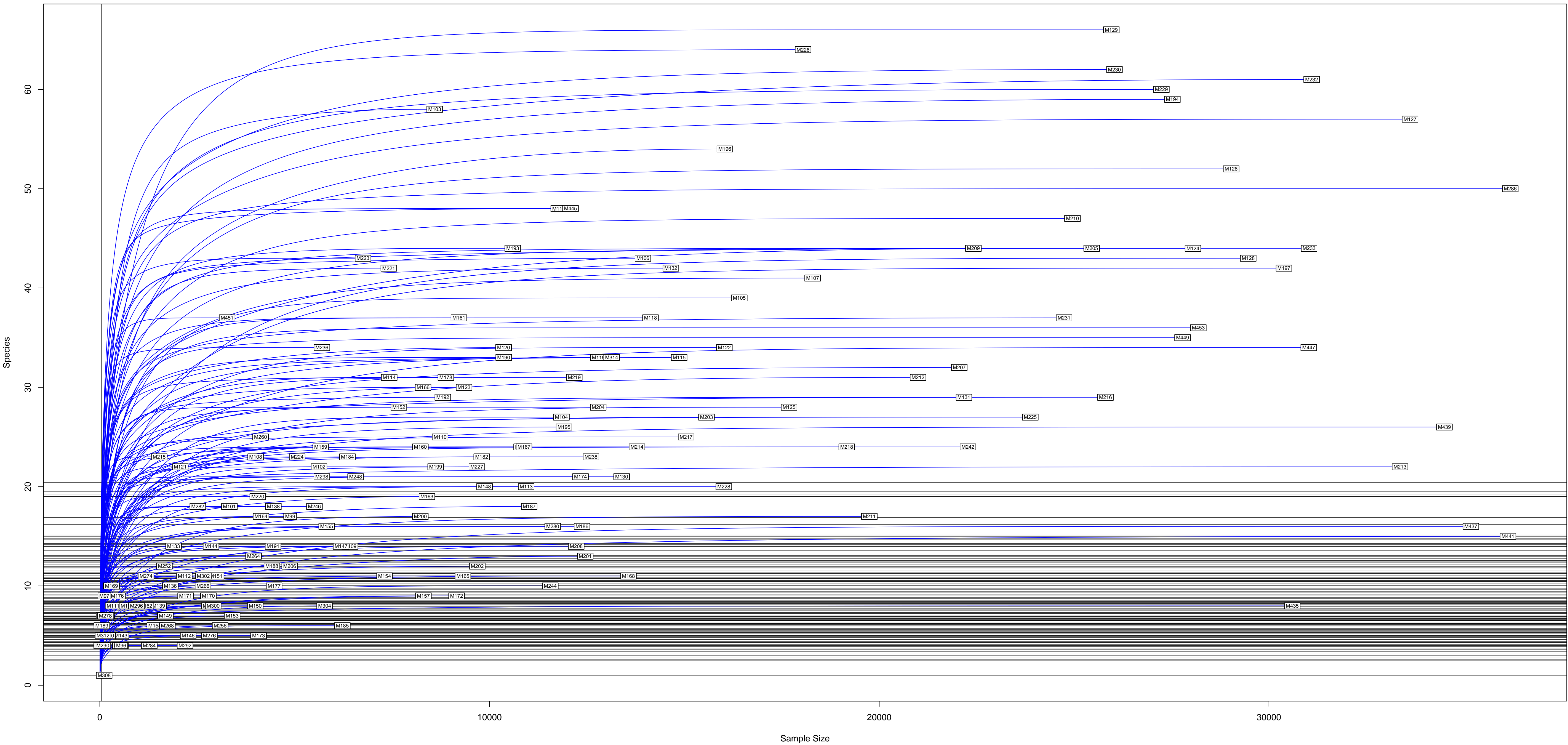

Supplement: S1 Fig — (PDF) [file pone.0298599.s001.pdf]

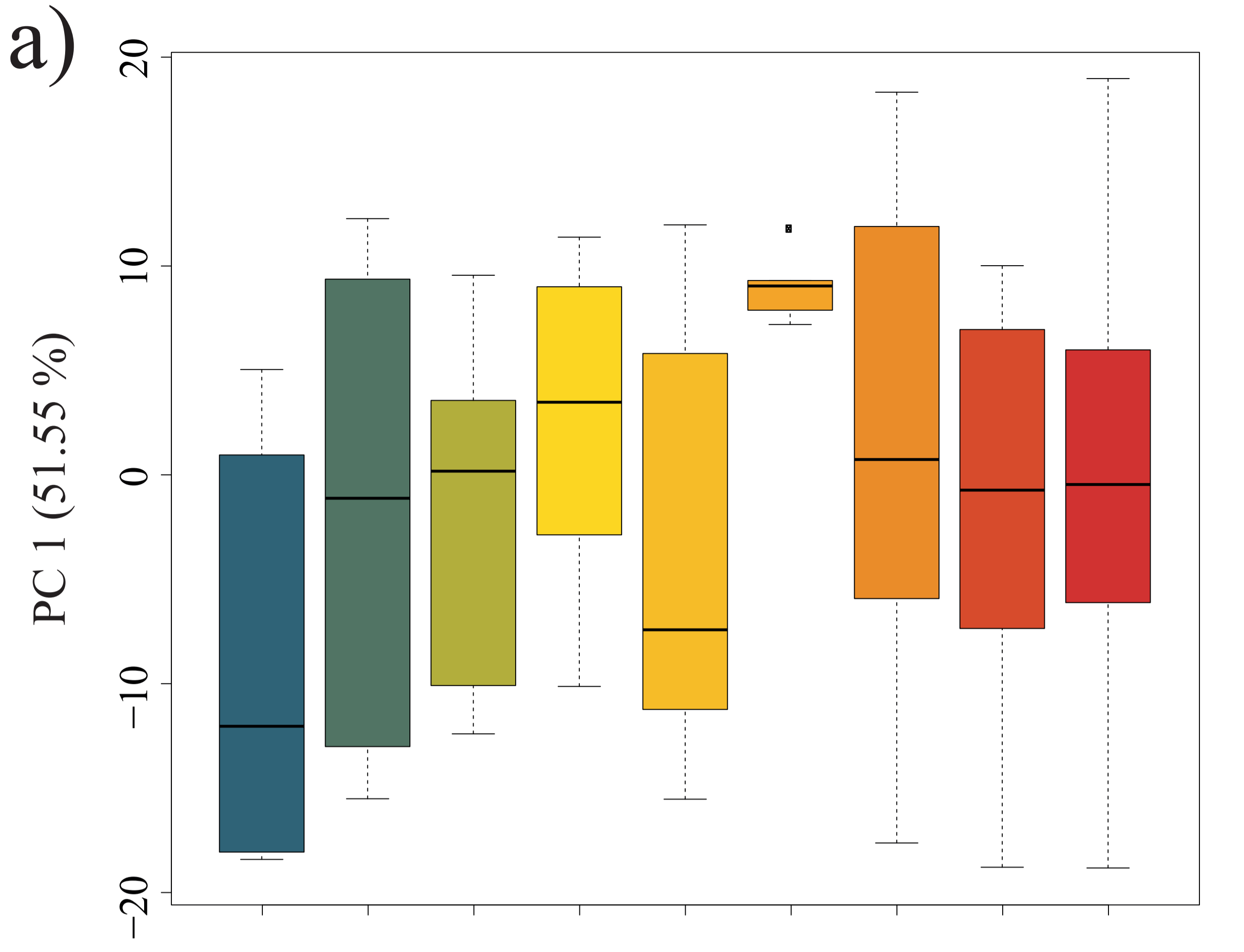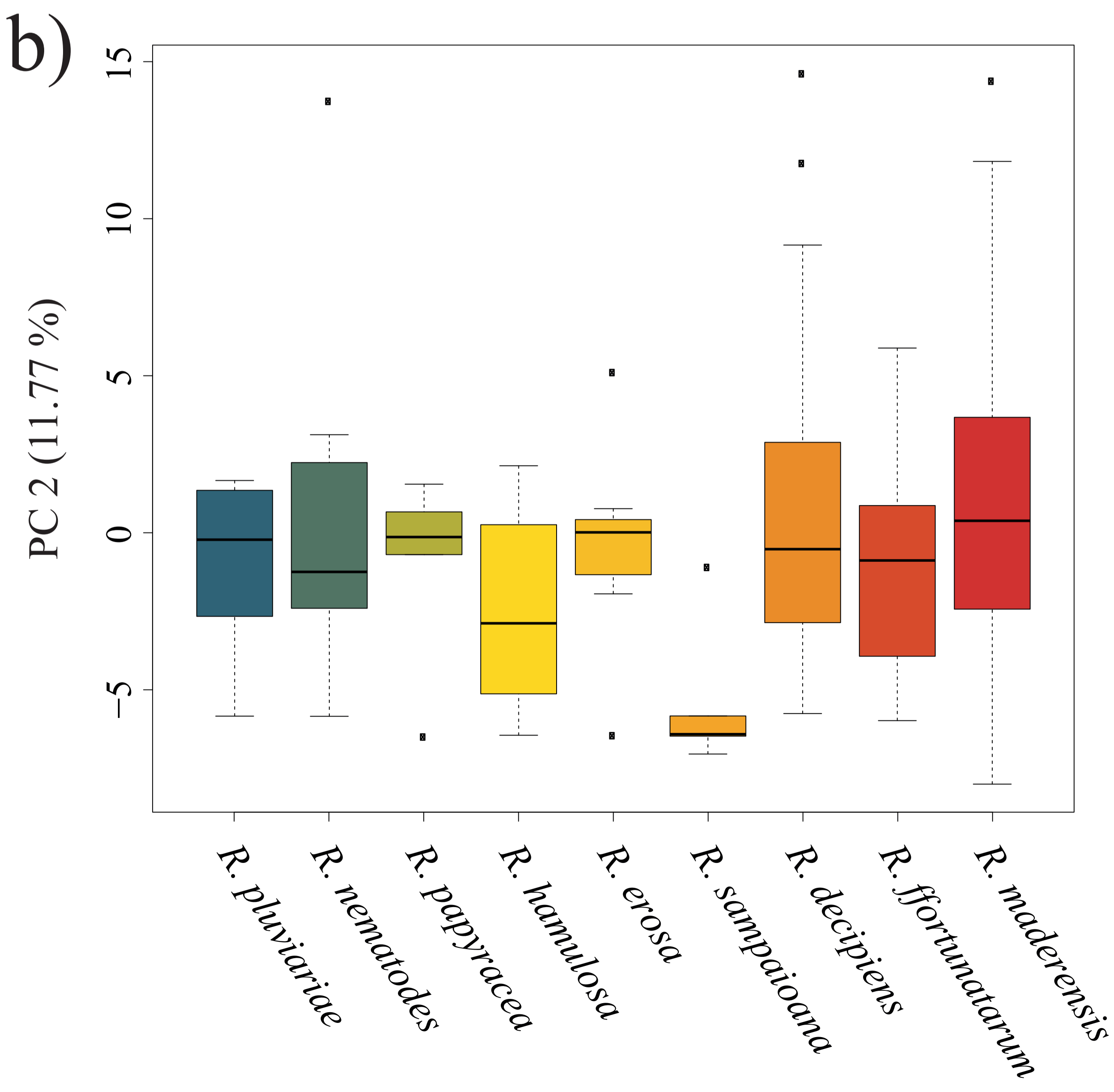

Supplement: S2 Fig — Boxplots show the coordinates of the studied specimens in the (a) first and (b) second principal components of the PCA computed from the 185 metabolic pathways predicted by PICRUST2 in which there where interspecific differences. (PDF) [file pone.0298599.s002.pdf]
